# The structure of *Phocaeicola vulgatus* sialic acid acetylesterase

**DOI:** 10.1107/S2059798322003357

**Published:** 2022-04-26

**Authors:** Hannah Scott, Gideon J. Davies, Zachary Armstrong

**Affiliations:** a Department of Chemistry, University of York, Heslington, York YO10 5DD, United Kingdom; b Department of Bioorganic Synthesis, Leiden University, Einsteinweg 55, 2333 CC Leiden, The Netherlands

**Keywords:** acetylesterases, SGNH fold, serine hydrolases, sialic acid acetylesterases, gut bacteria, carbohydrate metabolism, *Phocaeicola vulgatus*

## Abstract

Sialic acids terminate many N- and O-glycans and are widely distributed on cell surfaces. There are a diverse range of enzymes which interact with these sugars throughout the tree of life. They can act as receptors for influenza and specific betacoronaviruses in viral binding and their cleavage is important in virion release. Sialic acids are also exploited by both commensal and pathogenic bacteria for nutrient acquisition. A common modification of sialic acid is 9-*O*-acetylation, which can limit the action of sialidases. Some bacteria, including human endosymbionts, employ esterases to overcome this modification. However, few bacterial sialic acid 9-*O*-acetylesterases (9-*O*-SAEs) have been structurally characterized. Here, the crystal structure of a 9-*O*-SAE from *Phocaeicola vulgatus* (PvSAE) is reported. The structure of PvSAE was determined to resolutions of 1.44 and 2.06 Å using crystals from two different crystallization conditions. Structural characterization revealed PvSAE to be a dimer with an SGNH fold, named after the conserved sequence motif of this family, and a Ser–His–Asp catalytic triad. These structures also reveal flexibility in the most N-terminal α-helix, which provides a barrier to active-site accessibility. Biochemical assays also show that PvSAE deacetylates both mucin and the acetylated chromophore *para*-nitrophenyl acetate. This structural and biochemical characterization of PvSAE furthers the understanding of 9-*O*-SAEs and may aid in the discovery of small molecules targeting this class of enzyme.

## Abstract 2

The sialic acid acetylesterase from *P. vulgatus* was produced heterologously in *Escherichia coli*, purified and crystallized in two different crystal forms, from which structures at 1.44 and 2.06 Å resolution were obtained.

## 1. Introduction

**Figure 1:**
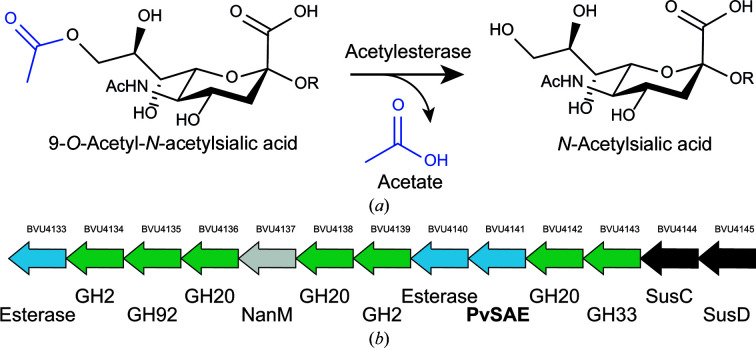
(*a*) Sialic acid acetylesterases remove the acetyl groups from various sialylated glycoproteins and glycolipids. (*b*) Genomic context of PvSAE from *P. vulgatus*. Locus tags are given above the arrows and the predicted annotation is given below. Genes that are predicted to be esterases are highlighted in blue, while glycoside hydrolases (GHs) are highlighted in green and SusC-like and SusD-like proteins are shown in black. Genes are not shown to scale.

Sialic acids are abundant monosaccharides that terminate glycolipids and glycoproteins on cell surfaces (Barnard *et al.*, 2020 ▸). As a result, they play key roles in a range of functions within humans, bacteria and viruses (Tortorici *et al.*, 2019 ▸). Sialic acids are also highly diverse, and are frequently 9-*O*-acetylated. In humans, 9-*O*-acetylation is regulated by CASD1 and sialic acid acetylesterase (SIAE), where CASD1 acetyl­ates and (SIAE) deacetylates sialic acid (Baumann *et al.*, 2015 ▸; Orizio *et al.*, 2015 ▸; Fig. 1 ▸ *a*). A key target molecule for SIAE is the 9-*O*-acylated GD3 (Neu5,9Ac_2_-α2,8-Neu5Ac-α2,3-Gal-β1,4-Glc-β1-ceramide); this ganglioside has an impact on a variety of biological functions. For example, elevated levels of 9-*O*-acetylsialic acid have been linked to apoptotic resistance by tumour cells, such as in medulloblastoma (a form of brain cancer; Mather *et al.*, 2019 ▸).

Viruses such as influenza C and specific betacoronaviruses (human OC43 and HKU1 coronaviruses) use 9-*O*-acetylated sialylglycans as receptors. To promote viral release after infection, these viruses use 9-*O*-acetylesterases (9-*O*-SAEs) as receptor-destroying enzymes (RDEs). The RDE for influenza C is the hemagglutinin esterase fusion protein (HEF) and that for betacoronaviruses is hemagglutinin esterase (HE). These enzymes also bind to 9-*O*-acetylsialic acid during viral entry. To date, HEFs and HEs from four betacoronaviruses have been structurally characterized and their 9-*O*-acetylesterase domains have SGNH hydrolase-like folds (Rosenthal *et al.*, 1998 ▸; Hurdiss *et al.*, 2020 ▸; Zeng *et al.*, 2008 ▸; Langereis *et al.*, 2012 ▸).

In bacteria, esterases play an important role in glycan foraging and catabolism of sialic acid in the gut (Robinson *et al.*, 2017 ▸). There is a high level of sialic acid diacetylation in humans, such as in the intestine distal column and rectum (Robinson *et al.*, 2017 ▸). However, sialidases encoded by bacteria cannot access diacetylated sialic acid. Therefore, bacteria employ esterases to make acetylated sialic acid more accessible for cleavage by sialidases. Carbohydrate-active esterases have been classified into several different families based on sequence homology (Drula *et al.*, 2022 ▸; Lenfant *et al.*, 2013 ▸) and have domain architectures such as α/β-hydrolase, (β/α)_7_-barrel and SNGH folds. As with HE and HEF, the structurally characterized 9-*O*-acetylsialic acid esterase NanS, encoded by enterohemorrhagic *Escherichia coli* O157:H7, also has an SGNH hydrolase fold (Rangarajan *et al.*, 2011 ▸).

The SGNH hydrolase superfamily was identified by Upton and Buckley through sequence comparisons of various motifs and was classified from members of the GDSL family (Upton & Buckley, 1995 ▸). GDSL esterases are named after the distinct GDSL motif near the N-terminus of the sequence that contains the catalytic nucleophile (Upton & Buckley, 1995 ▸). SGNH hydrolases have been defined by the structure of rhamnogalacturonan acetylesterase and classified with regard to the presence of Ser-Gly-Asn-His (SGNH) residues within four blocks of conserved sequences (I, II, III and V; Mølgaard *et al.*, 2000 ▸). These residues play important roles in the catalytic activity of the enzymes. The nucleophilic serine is found in block I of the sequences. The glycine and asparagine residues, within blocks II and III, respectively, stabilize the oxyanion in the oxyanion hole. The catalytic histidine within block V acts as a base by deprotonating the nucleophilic serine (Mølgaard *et al.*, 2000 ▸). Block V also contains an aspartic acid residue forming the third member of the Ser–His–Asp catalytic triad.

*Phocaeicola vulgatus* (previously known as *Bacteroides vulgatus*) is a commensal gut bacterium that has a cooperative role in the gut microbiome (Huang *et al.*, 2015 ▸). Sialidases encoded by several Bacteriodetes species cleave sialic acids from the intestinal tissue, which in turn has been shown to increase *E. coli* growth, resulting in ‘enterobacterial blooms’ which are apparent during inflammation (Huang *et al.*, 2015 ▸). A close homolog of PvSAE from *B. fragilis* (EstA; 81% identity) has been shown to have a role in the complex network of interactions involving polysaccharides in the gut. Robinson and coworkers developed further understanding of the role of *B. fragilis* in this network and highlighted the community scavenging of sialic acid in the gut (Robinson *et al.*, 2017 ▸). *E. coli* growth significantly increased in the presence of *B. fragilis* esterase with either *B. fragilis* or *B. thetaiotaomicron* sialidase, demonstrating how the microorganisms cooperate. Furthermore, PvSAE is found in a genomic locus that contains multiple glycoside hydrolases, including a sialidase (Fig. 1 ▸ *b*), that has synteny with loci from several Bacteroidetes including *P. massiliensis*, *B. fragilis* and *B. thetaiotaomicron* (Briliūtė *et al.*, 2019 ▸). The syntenic loci from *B. thetaiotaomicron* has recently been characterized as part of the sialylated N-glycan-degrading machinery of this host-associated bacterium (Briliūtė *et al.*, 2019 ▸).

The structure of 9-*O*-SAEs, including PvSAE, should provide insight into the function of sialic acid esterases and add to our understanding of host-glycan degradation. Furthermore, as this class of enzymes is also preserved in pathogens such as *Tanerella forsythia* (Albers *et al.*, 2021 ▸), investigating the structure of these enzymes should aid in the discovery of therapeutics for both bacterial and viral enzymes. Here, we show through SEC-MALLS that the 9-*O*-SAE from *P. vulgatus* (PvSAE) exists as a homodimer in solution. We also show through biochemical assays that PvSAE is a genuine SAE with activity on both chromogenic and natural mucins. The structure of PvSAE has previously been solved and deposited in the PDB (PDB entry 6njc). Here, we have solved the structure of PvSAE in two new crystal forms at 1.44 and 2.06 Å resolution, which reveal a previously unidentified conformational flexibility of this enzyme. We also show through docking studies that this enzyme may bind sialic acids.

## 2. Materials and methods

### 2.1. Enzyme production and purification

The gene encoding PvSAE (GenBank accession No. ABR41743.1) was predicted to contain a signal peptide with a cleavage site between amino acids 19 and 20 by *SignalP*5.0 (Almagro Armenteros *et al.*, 2019 ▸). A codon- optimized version of this gene with a His_6_ tag (MGSSHHHHHHGTAENLYFQG) in place of the signal peptide was synthesized and cloned into a pET-28 vector with NcoI and BamHI restriction sites by GenScript (Leiden, Netherlands). The plasmid was transformed into chemically competent *E. coli* BL21(DE3) Gold cells, which were then plated onto LB agar containing 50 µg ml^−1^ kanamycin. A single colony was used to inoculate 1 l LBE-5052 autoinduction medium (Studier, 2005 ▸) containing 50 µg ml^−1^ kanamycin, which was incubated overnight with shaking at 300 rev min^−1^ and 37°C. Expression cultures were harvested by centrifugation (5000*g*, 30 min, 4°C). The pellets were resuspended in 25 ml resuspension buffer (20 m*M* HEPES, 200 m*M* NaCl pH adjusted to 7.4 with NaOH) and stored at −70°C until use.

Frozen pellets were thawed and 1.8 ml buffer *B* (20 m*M* HEPES, 500 m*M* imidazole, 200 m*M* NaCl, pH adjusted to 7.4 with HCl) was added with lysozyme (4 mg) and DNase I (1 mg). The volume was increased to 50 ml with buffer *A* (20 m*M* HEPES, 20 m*M* imidazole, 200 m*M* NaCl, 1 m*M* DTT, pH adjusted to 7.4 with HCl). After warming to room temperature, the cell suspension was vortexed and sonicated on ice for 1 min 40 s (1 s sonication, 2 s rest) in a sonicator (Qsonica) set to 40% amplitude. The lysed cells were centrifuged (18 000*g*, 30 min, 4°C) and the supernatant was directly loaded onto a 5 ml HisTrap Excel column (GE Healthcare). The bound protein was washed with ten columns of buffer *A* and then subsequently eluted using a 0–100% linear gradient of buffer *B* over 20 column volumes. Fractions containing PvSAE were determined by SDS–PAGE and the peak fractions were pooled. Some precipitate was present, so the pooled fractions were centrifuged and filtered (0.22 µm filter). The filtrate was concentrated to a final volume of 2 ml using a Vivaspin 10 kDa concentrator (GE Healthcare) and further purified by gel filtration (HiLoad 16/600 Superdex 75 pg) in buffer *C* (20 m*M* HEPES, 200 m*M* NaCl, 1 m*M* DTT, pH adjusted to 7.4 with NaOH) at a flow rate of 1 ml min^−1^. Fractions containing PvSAE were determined using SDS–PAGE and the peak fractions were pooled and concentrated using a Vivaspin 10 kDa concentrator (GE Healthcare). The concentrated protein was washed with buffer *C* and diluted to 50 mg ml^−1^. Aliquots (40 µl) were flash-frozen in liquid nitrogen and stored at −70°C until use. Protein concentrations were determined spectrophotometrically using a calculated *A*_280_ extinction coefficient of 32 890 *M* ^−1^ cm^−1^. This purification yielded 45.6 mg protein per litre of culture. Macromolecule-production information is summarized in Table 1 ▸.

### 2.2. SEC-MALLS

Experiments were conducted on a system comprising a Wyatt HELEOS-II multi-angle light-scattering detector and a Wyatt rEX refractive-index detector linked to a Shimadzu HPLC system (SPD-20A UV detector, LC20-AD isocratic pump system, DGU-20A3 degasser and SIL-20A autosampler). Work was conducted at room temperature (20 ± 2°C). The sample-injection volume was at 100 µl at a protein concentration of 2 mg ml^−1^. The sample was separated on a Superdex 200 Increase 10/300 GL column (GE Healthcare) using 20 m*M* HEPES, 200 m*M* NaCl, pH adjusted to 7.4 with NaOH (0.2 µm filtered) as buffer. A further 0.1 µm filter was present in the flow path. Shimadzu *LabSolutions* software was used to control the HPLC and the *ASTRA* 7 software was used for the HELEOS-II and rEX detectors. The data were analysed using the *ASTRA* 7 software. Molecular masses were estimated using the Zimm fit method with degree 1 (Zimm, 1945 ▸). A value of 0.182 was used for the protein refractive-index increment (d*n*/d*c*).

### 2.3. Crystallization

Initial crystallization conditions were identified using a range of commercial crystallization screens in 96-well format plates, including PEG/Ion (Hampton Research), JCSG+ (Molecular Dimensions), The AmSO4 Suite (Qiagen), PACT (Molecular Dimensions), Index (Hampton Research) and Morpheus (Molecular Dimensions). The condition 0.2 *M* sodium malonate/malonic acid pH 5.0, 20%(*w*/*v*) polyethylene glycol 3350 with His_6_-PvSAE at 30 mg ml^−1^ in a 150 nl:150 nl ratio yielded thin rod-like crystals and this condition was further optimized. The optimized crystals were grown in MRC Maxi 48-well plates using the sitting-drop vapour-diffusion method at 20°C with 0.2 *M* sodium malonate/malonic acid pH 4.5, 18%(*w*/*v*) polyethylene glycol 3350 and His_6_-PvSAE at 30 mg ml^−1^ in buffer *C* with a 400 nl:400 nl protein:well solution ratio; this condition will be referred to as Apo I. Crystals were soaked in well solution consisting of 0.2 *M* sodium malonate/malonic acid pH 4.5, 18%(*w*/*v*) polyethylene glycol 3350, 15%(*w*/*v*) ethylene glycol before flash-cooling in liquid nitrogen.

Crystals were also observed in the Morpheus screen and were collected without cryoprotectant from a condition consisting of 50% precipitant mix 4 [25%(*w*/*v*) PEG 1000, 25%(*w*/*v*) PEG 3350, 25%(*v*/*v*) MPD], 0.03 *M* sodium nitrate, 0.03 *M* sodium phosphate, 0.03 *M* ammonium sulfate, 0.1 *M* MOPS/HEPES–Na pH 7.5 (prepared according to Gorrec, 2009 ▸), with His_6_-PvSAE at 30 mg ml^−1^ in buffer *C*. This condition will be referred to as Apo II. The crystals were harvested directly from the crystallization drop before being flash-cooled in liquid nitrogen. Crystallization information is summarized in Table 2 ▸.

### 2.4. Data collection, processing, structure solution and refinement

All data sets were collected on the I03 beamline at Diamond Light Source (DLS) and were integrated using the *DIALS* pipeline in *xia*2. All other calculations were carried out using the *CCP*4 suite (Winn *et al.*, 2011 ▸). Data reduction was performed using *AIMLESS* (Evans & Murshudov, 2013 ▸) and the data for Apo I and Apo I crystals were processed to resolutions of 1.44 and 2.06 Å, respectively. The structures were solved by expert-mode molecular replacement with *Phaser* (McCoy *et al.*, 2007 ▸) using the coordinates of BVU4141 from *P. vulgatus* as a search model (PDB entry 6njc; Midwest Center for Structural Genomics, unpublished work). The models were corrected and completed manually with multiple rounds of model building using *Coot* (Emsley *et al.*, 2010 ▸) and refined using *REFMAC*5 (Murshudov *et al.*, 2011 ▸). There was suspected twinning in the structure obtained from the Apo II condition and this was confirmed by an *L*-test score of 0.42. To take twinning into account, twin refinement was used for the Apo II structure in *REFMAC*5 once the *R*_free_ fell below 40%.

Water molecules for both models were added using *REFMAC*5 (Murshudov *et al.*, 2011 ▸) and were manually inspected after refinement using *Coot* (Emsley *et al.*, 2010 ▸). The final refinement statistics can be found in Table 3 ▸. Data-collection and processing statistics are summarized in Table 3 ▸ and refinement statistics are summarized in Table 4 ▸.

### 2.5. Michaelis–Menten kinetics

Hydrolysis of chromogenic *para*-nitrophenyl acetate (pNP-Ac) was carried out in a 96-well plate at 25°C. Reactions were carried out in a total volume of 100 µl. Each reaction contained 10 n*M* PvSAE, 100 m*M* phosphate–citrate buffer pH 7.0 and 1 m*M* to 50 µ*M* pNP-Ac in 1% DMSO. Reactions were initiated by the addition of 10 µl 100 n*M* enzyme to a reaction containing 90 µl water, buffer and pNP-Ac. All kinetic assays were completed in triplicate.

Reactions were measured using a Clariostar microplate reader (BMG Labtech; λ_ex_ = 405 nm). The absorbance of released pNP was measured continuously over 15 min. The concentration of pNP released was calculated using a calibration curve of pNP standards (100 µ*M* to 1 µ*M*) present on the same plate. Rates of substrate hydrolysis were determined by a linear fit of initial data points. Plots of the initial rate (*v*_o_) *versus * the substrate concentration were fitted with nonlinear regression using *OriginPro* 2021 (OriginLab) to the hyperbolic equation *v*_o_ = *k*_cat_[E][S]/(*K*_m_ + [S]) to determine *K*_m_ and *k*_cat_ (Table 5 ▸). The error values given for *K*_m_ and *k*_cat_ are fitting errors determined in *OriginPro* when fitting all data. The error values given for the specificity constant (*k*_cat_/*K*_m_) were propagated from the error determined for *K*_m_ and *k*_cat_ using the formula

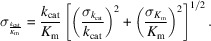




### 2.6. Mucin activity assays

The hydrolysis of acetylated sialic acids present in mucin was assessed using bovine submaxillary mucin (Millipore, Lot No. 3716979). Purified PvSAE was added to a final concentration of 1 µ*M* or 100 n*M* to a 100 µl reaction consisting of 6.25 mg ml^−1^ bovine submaxillary mucin, 20 m*M* HEPES, 100 m*M* NaCl pH 7.0 (adjusted with NaOH). The samples were incubated at 37°C for 1 h with shaking at 800 rev min^−1^. The concentration of acetate in each sample was quantified using a manual acetic acid assay kit (Megazyme, K-ACET) according to the manufacturer’s instructions. HEPES buffer was used in place of phosphate buffer for this assay as phosphate interferes with coupled enzyme activity in the acetic acid assay kit. An acetate standard curve (0–800 µ*M* acetate) in the presence of 6.25 mg ml^−1^ bovine submaxillary mucin was used to calibrate the acetate concentration. All measurements were performed in at least quadruplicate.

### 2.7. Molecular docking

Molecular docking of 9-*O*-acetylsialic acid into the crystal structure of PvSAE was performed with *AutoDock Vina* (Trott & Olson, 2010 ▸). The ligand coordinates were made using *AceDRG* (Long *et al.*, 2017 ▸) and molecular charges were computed with *AutoDock* (Morris *et al.*, 2009 ▸). The ligand was docked into a grid box (30 × 20 × 20 Å) centred on the catalytic nucleophile. A search was performed on each of the active sites for the Apo I and Apo II structures. An exhaustiveness of 30 was used to compute 20 docking positions for each active site. The docked position with the best calculated energy and with the acetyl group positioned in the active site was used for further analysis.

## 3. Results and discussion

### 3.1. Purification and biochemical characterization

*P. vulgatus* 9-*O*-acetylsialic acid esterase (PvSAE) was produced in *E. coli* BL21(DE3) Gold cells. The protein contained an N-terminal His_6_ tag and was purified using immobilized nickel-affinity chromatography followed by size-exclusion chromatography. The protein eluted earlier than expected for a monomer from a calibrated size-exclusion column. To determine whether the protein was a multimer in solution, it was subjected to SEC-MALLS (size-exclusion chromatography–multi-angle laser light scattering). A single peak was observed in the chromatogram with a calculated molar mass of 51.6 kDa, indicating that PvSAE forms a dimer in solution.

**Figure 2:**
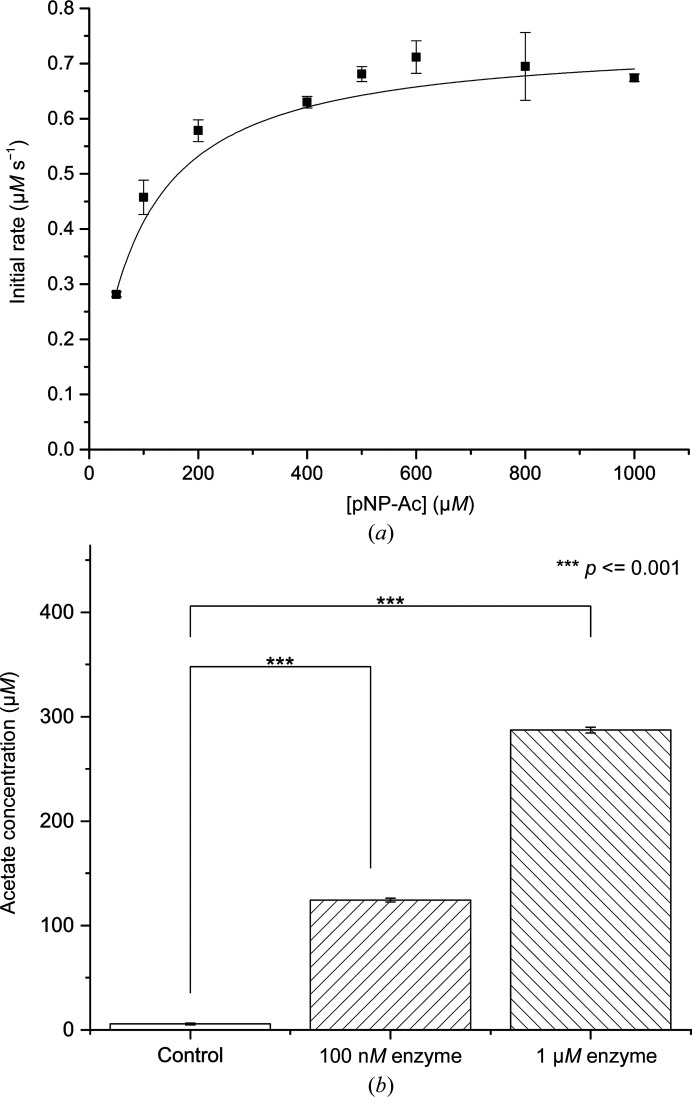
(*a*) Michaelis–Menten kinetic plot for hydrolysis of pNP-Ac by PvSAE. Rates were established using a pNP standard curve. Experiments were performed in triplicate. (*b*) Activity of PvSAE on bovine submaxillary mucin. Acetate release from 6.25 mg ml^−1^ bovine submaxillary mucin was quantified after 1 h of incubation at 37°C with a buffer control or 100 n*M* or 1 µ*M* PvSAE.

To gain further insight into the action of PvSAE, we investigated the activity of PvSAE on both a synthetic substrate and a natural mucin. We first tested the activity of PvSAE on the activated, chromogenic substrate *para*-nitrophenyl acetate (pNP-Ac). This substrate has previously been used to detect 9-*O*-SAE activity for *E. coli* NanS, *B. fragilis* EstA and *Tannerella forsythia* NanS (Rangarajan *et al.*, 2011 ▸; Nakayama-Imaohji *et al.*, 2012 ▸; Albers *et al.*, 2021 ▸). pNP-Ac hydrolysis was monitored at pH 7.0 to limit nonspecific acid- or base-catalyzed hydrolysis. The catalytic constants determined from a Michaelis–Menten plot of initial rates (Fig. 2 ▸ *a*), revealed a high specificity rate constant (*k*_cat_/*K*_m_ = 930 ± 70 m*M* ^−1^ s^−1^) for this non-natural substrate, with rates slightly higher than that determined for the bifunctional NanS from *T. forsythia* (*k*_cat_/*K*_m_ = 434 m*M* ^−1^ s^−1^; Albers *et al.*, 2021 ▸).

To examine the activity of PvSAE on a more natural substrate, we assessed its activity on bovine submaxillary mucin (BSM). BSM is purified directly from the submaxillary gland, allowing access to the natural substrate before it has been acted on by commensal microbes. As such, it contains several percent sialic acid by weight and is highly acetylated. We incubated either 100 n*M* or 1 µ*M* PvSAE with BSM for 1 h at 37°C. At both enzyme concentrations PvSAE showed a significant release of acetate when compared with a buffer-only control (Fig. 2 ▸ *b*). This indicates that PvSAE is indeed a genuine sialic acid esterase that is capable of action on natural substrates.

### 3.2. Protein crystallization and structure solution

Purified PvSAE crystallized in two different forms (Apo I and Apo II; see Table 2 ▸ for crystallization conditions). A crystal from the Apo I condition diffracted to a resolution of 1.44 Å and had *P*12_1_1 symmetry, while a crystal from the Apo II condition diffracted to 2.06 Å resolution and had *P*6_1_ symmetry. Both structures were solved by molecular replace­ment using a structure of PvSAE at 1.9 Å resolution deposited in the PDB (PDB entry 6njc; Midwest Center for Structural Genomics, unpublished work). The unit cells of these crystals contained four and eight protomers for the Apo I and Apo II forms, respectively. These protomers were arranged as dimers in both crystal forms, recapitulating the dimeric form observed in solution.

**Figure 3:**
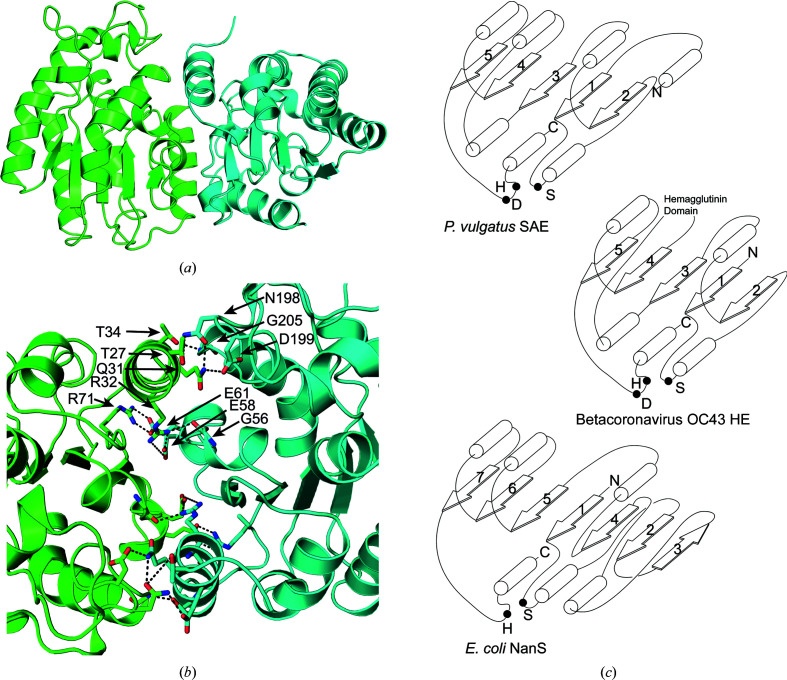
(*a*) Apo I PvSAE dimer structure shown in cartoon representation. (*b*) PvSAE dimer interface. Residues forming salt bridges and hydrogen bonds between two protomers are shown as sticks. Hydrogen bonds and salt bridges are shown as black dashed lines. The residues from one of the two symmetrical interaction interfaces are labelled. The two protomers are coloured green and cyan. (*c*) Domain organization of sialic acid esterases. β-­Strands are numbered according to their primary-sequence positions. The β-strands present in the hemagglutinin domain of OC43 HE have been omitted for simplicity. The catalytic serine (S), histidine (H) and aspartate (D) are labelled. The N-terminus and C terminus are denoted N and C, respectively. Structural elements are not drawn to scale.

The PvSAE Apo I and Apo II structures displayed a near-complete overlap when aligned [root-mean-square deviation (r.m.s.d.) of 0.18 Å], with the secondary structures in the same conformation. The only major difference between these structures was a small region of the most N-terminal α-helix, which is discussed below. The individual protomers have a fold with a central five-stranded parallel β-sheet surrounded by α-helices (Fig. 3 ▸ *a*), which is typical of an SNGH type I domain. This domain architecture is more similar to that of beta­coronavirus HEs (Bakkers *et al.*, 2017 ▸) than to the domain organization observed for *E. coli* NanS (Rangarajan *et al.*, 2011 ▸; Fig. 3 ▸ *c*). Betacoronavirus HEs also contain a central five-stranded parallel β-sheet surrounded by α-helices. However, unlike PvSAE these viral enzymes contain a hemagglutinin domain inserted between the third and fourth β-strands of the β-sheet. The structure of *E. coli* NanS, in contrast, contains a seven-stranded β-sheet with six parallel β-strands and one antiparallel β-strand (Rangarajan *et al.*, 2011 ▸), as is typical of SNGH type II domains. The suspected serine nucleophile of PvSAE, Ser52, is located at the beginning of the second α-helix, following the first β-strand from the N-terminus, a position that is preserved in both *E. coli* NanS and beta­coronavirus HEs (Fig. 3 ▸ *c*). The suspected catalytic histidine and aspartic acid, His202 and Asp199, are located on an Ω loop which is followed by the most C-terminal α-helix.

As stated above, each of the protomers is present in a dimer in both of the crystal structures determined here and in the previously determined structure of PvSAE. These dimers have *C*2 rotational symmetry, and interactions between the two interfaces were examined using *PISA* (Krissinel & Henrick, 2007 ▸). The dimer interface is mainly composed of hydrophilic residues that form salt bridges and hydrogen bonds (Fig. 3 ▸ *b*). A majority of these interactions are formed between residues on the most N-terminal α-helix (residues 27–34) and residues on the Ω loop containing the catalytic histidine and aspartic acid (residues 198–205) (Fig. 3 ▸ *b*). This interaction also involves the aspartic acid Asp199 (a constituent of the Asp–His–Ser catalytic triad), which forms a hydrogen bond to Gln31 in the neighbouring protomer. We expect that the dimeriz­ation of PvSAE observed here maintains the correct positioning of the catalytic residues. Dimerization results in a loss of 1300 Å^2^ of solvent-accessible surface for each monomer and leads to the formation of a 15 Å deep cleft. This cleft contains both active sites, which face each other and are separated by 15 Å, determined as the distance between the proposed serine nucleophiles.

The most significant difference between the Apo I and Apo II structures was the positioning of the most N-terminal residues (residues 24–27) of the first α-helix. In the Apo II structure and the previously solved structure of PvSAE (PDB entry 6njc) this α-helix extends above the active-site cleft, restricting the entrance to the active-site cleft to approximately 8 Å (Supplementary Fig. S1). In the Apo I structure residues 24–27 of one of the protomers in the homodimer are no longer part of this α-helix structure, but instead form a loop projecting out of the active site. This conformational change is supported by a hydrogen bond between the hydroxyl of Tyr25 and the carboxylic acid of Asp83 from the neighbouring protomer. The result of this change in conformation is that one active site is more accessible and has an ‘open’ conformation, while the other is still ‘blocked’ by the N-terminal α-helix (Supplementary Fig. S1). Although the observed conformational change may be an artefact of the crystallization conditions, this change indicates that the residues present in the N-terminus have inherent plasticity. We suspect that this conformational change may be important for the accommodation of large, complex glycans that contain acetylated sialic acid.

### 3.3. Structural comparisons and active site

To investigate proteins with structural homology to PvSAE, a search was performed using the *DALI* server (Holm, 2020 ▸). This further supported PvSAE as a member of the SGNH family, as the search revealed that PvSAE shares low amino-acid sequence identity, but high structural similarity, with members of the SGNH family (Supplementary Fig. S2). Further comparison of the sequences of the top hits showed conservation of Ser52, Gly75, Asn103, His202 and Asp199. It was confirmed that the catalytic triad corresponds to Ser52, His202 and Asp199, with Gly75 and Asn103 responsible for forming the oxyanion hole. The residues within the conserved blocks of sequences common to the SGNH superfamily are also present. However, the block I sequence varied, with a GNS motif (Gly50, Asn51, Ser52) compared with the classic GDS motif (Supplementary Fig. S2). The presence of aspara­gine in the place of aspartate in PvSAE, however, does not change the positioning of this amino acid when compared with the structure of the top *DALI* result with a GDS motif (putative platelet-activating factor from *Streptococcus pneumoniae*; PDB entry 2hsj; Supplementary Fig. S2). The remaining three sequence blocks (II, III and V) were conserved across the *DALI* hits. These blocks contain the conserved glycine (block II), the conserved asparagine in a G*X*N motif (block III) and the catalytic serine and histidine in a D*XX*H motif (block V).

**Figure 4:**
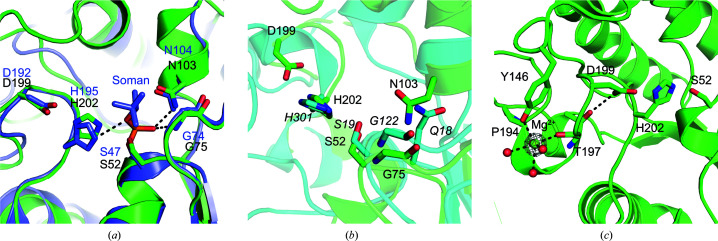
Active-site structure of PvSAE. (*a*) Active-site overlay of Apo I PvSAE and pAF-AH (PDB entry 3dt9), with active-site residues and soman inhibitor shown as sticks. PvSAE residues are labelled in black and pAF-AH residues are labelled in blue. Polar contacts between the soman inhibitor and active-site residues are displayed with black dashed lines. pAF-AH and soman inhibitor C atoms are coloured purple, while PvSAE C atoms are coloured green. (*b*) Active-site overlay of PvSAE and NanS (PDB entry 3pt5; Rangarajan *et al.*, 2011 ▸), with active-site residues shown as sticks. NanS residues are labelled in italics and NanS is coloured cyan. (*c*) Mg^2+^ coordination with surrounding electron density. The electron density is σ_A_-weighted 2*F*_o_ − *F*_c_ density contoured at 2σ (0.65 e^−^ A^−3^) and was rendered using *PyMOL*. The sphere scale is set at 0.25. The PvSAE catalytic triad is also shown.

Both the Apo I and Apo II PvSAE structures had an Mg^2+^ metal ion in close proximity to the active site. This ion is also observed in the previously reported structure of PvSAE (PDB entry 6njc). The electron density surrounding Mg^2+^, the bond lengths and the octahedral coordination to six electron donors support this annotation. The metal was likely to be incorporated into the structure during the production of the enzyme. Mg^2+^ interacts with the carbonyl groups of Pro194 and Thr197, the side-chain hydroxyl group of Tyr146 and three waters. The Thr197 residue, in turn, is positioned to form a hydrogen bond to the catalytic aspartate (Asp199) (Fig. 4 ▸ *c*).

One of the top *DALI* hits for the structure of PvSAE was PAF-AH (brain platelet-activating factor acetylhydrolase). A structure of PAF-AH (PDB entry 3dt9) contains the covalent oganophosphorus inhibitor soman bound to the nucleophilic active-site serine (Epstein *et al.*, 2009 ▸). Overlay of PvSAE and pAF-AH displayed good overlap between the active-site residues (r.m.s.d. of 0.37 Å for residues within 8 Å of the nucleophilic serine), with the residues in similar conformations (Fig. 4 ▸ *a*). This suggests that organophosphorus inhibitors may also be active against PvSAE.

All of the top five *DALI* hits are homodimeric structures, as seen for PvSAE. As in PvSAE, the dimer interface in these five structures involves the N-terminal α-helix. However, the N-terminal α-helix does not interact with the Ω loop that contains the catalytic histidine in all of these structures (Supplementary Fig. S3). In three of these structures (PDB entries 3p94, 4ppy and 4iyj) the N-terminal α-helix interacts with residues present in the third and fourth α-helices, which in turn interact with the third and fourth α-helices on the neighbouring protomer. This results in more exposed active sites that are not part of the same active-site cleft. The dimeric structure of *S. pneumonia* putative platelet-activating factor (PDB entry 2hsj) is more similar to PvSAE, with the dimer interface between the N-terminal α-helix and the C-terminal α-helix. However, in this structure the N-terminal α-helix does not involve the Ω loop. The structure of pAF-AH (PDB entry 3dt9) also forms a dimer, with an interface composed of salt bridges formed by hydrophilic residues between the loop containing the catalytic histidine and aspartate and the N-terminal α-helix (Epstein *et al.*, 2009 ▸). The angle of this interaction interface, however, differs from that seen for PvSAE, resulting in a deeper active-site cleft than is observed for PvSAE.

The 9-*O*-SAE NanS from *E. coli* has a SGNH type II fold with catalytically active residues Ser19 and His301. This enzyme, however, lacks the catalytic aspartate residue from the catalytic triad. This interaction may be compensated for by a more remote carboxylate, the action of which is mediated through an ordered water molecule (Rangarajan *et al.*, 2011 ▸). Overlay of the active site of NanS and PvSAE (r.m.s.d. of 0.32 Å for an 8 Å sphere surrounding the catalytic serine) reveals that the catalytic histidine and serine residues occupy a similar position, despite the absence of a catalytic acid in NanS (Fig. 4 ▸ *b*). The oxyanion hole of NanS contains a glycine, as seen in PvSAE. However, in NanS the other constituent of the oxyanion hole is Gln18, as opposed to Asn103 in PvSAE (Fig. 4 ▸ *b*). Unlike PvSAE, *E. coli* NanS forms a monomer in solution, indicating that homodimerization is not conserved for 9-*O*-SAEs. The monomeric nature of *E. coli* NanS is likely to be due to the absence of the most N-terminal α-helix, which forms a large part of the dimer interface in PvSAE (Fig. 3 ▸ *c*).

EstA from *S. pneumoniae* has also been identified as a 9-*O*-SAE (Kahya *et al.*, 2017 ▸). This enzyme displays a canonical α/β-hydrolase topology with a Ser–His–Asp catalytic triad (Kim *et al.*, 2008 ▸). *S. pneumoniae* EstA, however, is not an SGNH hydrolase as the catalytic triad residues (Ser120–His231–Asp202) do not align with the conserved blocks typical of an SGNH hydrolase. The active site of EstA also differs from that of an SGNH hydrolase, with the backbone N atom from two methionine residues (Met45 and Met121) forming the oxyanion hole (Kim *et al.*, 2008 ▸), whereas in PvSAE the backbone N atom of a glycine (Gly75) and the amide side chain of an asparagine (Asn103) form the oxyanion hole (Supplementary Fig. S4).

**Figure 5:**
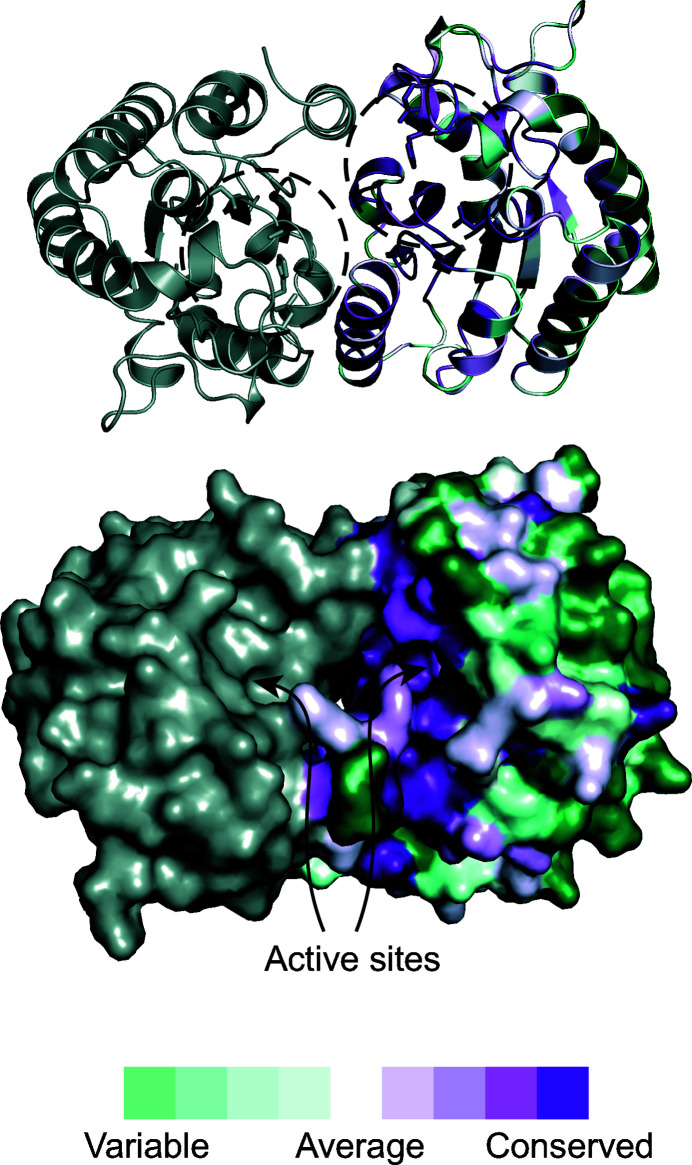
Conservation of the Apo I PvSAE surface. Active-site residues (Ser52, His202, Asp199, Asn103 and Gly75) are shown as sticks in the ribbon representation of Apo I PvSAE. Chain *A* is coloured by sequence conservation and chain *B* is coloured grey. Figures were prepared with *ConSurf* (Ashkenazy *et al.*, 2016 ▸) using the top 500 *BLAST* hits (Altschul *et al.*, 1990 ▸) to the protein sequence of PvSAE. The figure was generated using *PyMOL* (Schrödinger).

To further investigate the phylogenetic conservation of amino acids, conservation scores were determined using *ConSurf* (Ashkenazy *et al.*, 2016 ▸) and mapped onto the PvSAE structure. This revealed the absolute conservation of residues in the active site (Fig. 5 ▸). There was also absolute conservation of the interface amino-acid residues involved in forming salt bridges between the two dimers, namely Arg32, Arg71, Glu58 and Glu61. The central five-stranded β-sheet was relatively well conserved (*ConSurf* scores of 6–9), with only Lys33 displaying low conservation (*ConSurf* score of 2). The level of conservation between the α-helices varied; helices distal to the active site and protein interface, Ala111–Glu128 and Asn150–Glu173, displayed increased variability. Loops at the protein surface were also poorly conserved, in contrast to those near the active site. The level of conservation for the Mg^2+^-binding site varied. Thr197, which may interact with the active site, was relatively well conserved (*ConSurf* score of 7). Conversely, Tyr146 and Pro194 displayed poor conservation (*ConSurf* scores of 1 and 2, respectively).

**Figure 6:**
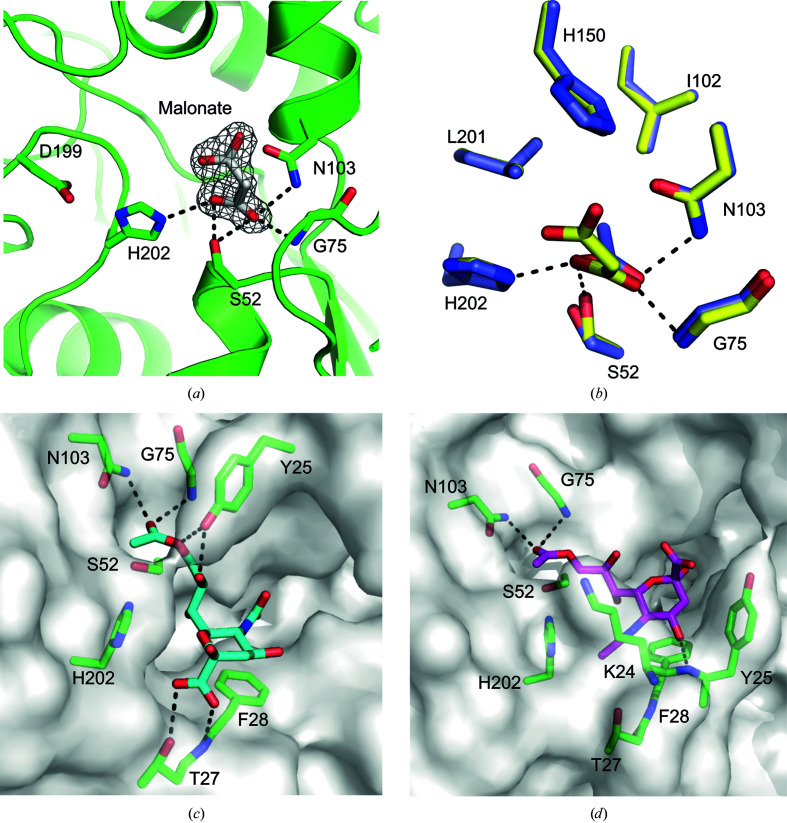
(*a*) Apo I PvSAE complex with malonate. Active-site residues and malonate are shown as sticks. The electron density surrounding malonate is σ_A_-­weighted 2*F*_o_ − *F*_c_ density contoured at 1σ (0.33 e^−^ A^−3^). (*b*) Superposition of active sites containing malonate and acetic acid. The Apo I structure, containing malonate, is shown with C atoms coloured green. PDB entry 6njc, containing acetate, is shown with C atoms coloured blue. (*c*) 9-*O*-Acetylsialic acid docked into the ‘open’ active site of the Apo I structure and (*d*) into the ‘blocked’ active site of Apo I. Polar interactions within 3.2 Å are shown as black dashed lines in all panels.

The high resolution of the Apo I structure allowed the identification of a malonate molecule in the active site of PvSAE. Malonate is positioned such that a carboxylate functional group is within hydrogen-bonding distance of the oxyanion-hole residues Asn103 and Gly75 (Fig. 6 ▸ *a*). This mirrors the positioning of acetate in the previously determined structure of PvSAE, where the acetate interacts directly with the oxyanion hole (Fig. 6 ▸ *b*). Automated docking of 9-*O*-acetylsialic acid into each active site showed that this substrate could fit into the active sites of both the Apo I and Apo II structures, with the 9-acetyl group correctly positioned for catalysis (Fig. 6 ▸ and Supplementary Fig. S6). As the most N-terminal α-helix of the Apo I structure has different conformations, the two active sites accommodate the modelled 9-*O*-acetylsialic acid in a different conformation. In the ‘open’ active site, the 9-*O*-acetylsialic acid is positioned such that the 2-position projects towards the bulk solvent, indicating that this site could accommodate larger glycans. There also appears to be space for both acetylation at the 7-position and an *N*-glycolyl modification at the 5-position, both of which are modifications that occur in animal mucins (Park, 2019 ▸). The carboxylate on the modelled ligand is within hydrogen-bonding distance of the backbone N atom of Phe28, which only becomes accessible once the N-terminal α-helix has adopted the ‘open’ conformation. This interaction with the carboxylate contrasts with the interaction seen in viral sialic acid esterases, which employ an active-site arginine as an interaction partner with the carboxylate (Rosenthal *et al.*, 1998 ▸). Docking into the ‘blocked’ site of the Apo I structure resulted in a ligand conformation with the 2-position projecting towards the N-terminal α-helix, suggesting that this active site could only accommodate monomeric substrates. A similar conformation to the blocked active site was observed in the Apo II structure (Supplementary Fig. S6).

## 4. Conclusion

9-*O*-Acetyl-*N*-acetylsialic acid esterases are found throughout the tree of life from viruses to bacteria and animals. Examination of the 9-*O*-SAE from the human commensal bacterium *P. vulgatus* (PvSAE) adds to our growing understanding of the structure and mechanisms underpinning this class of enzymes. Three-dimensional structures of PvSAE determined by X-ray crystallography reveal the homodimerization of PvSAE and the molecular architecture of the SGNH active site. This revealed a canonical Ser–His–Asp catalytic triad encompassed in a binding cleft formed through dimerization. Additionally, the conformational flexibility of the N-terminal α-helix appears to mediate the accessibility of the active cleft to large oligosaccharide substrates. The structural analysis of PvSAE also contributes to our understanding of other predicted SGNH hydrolases that are yet to be solved, such as human SIAE. Looking forwards, the structural analysis of 9-*O*-SAE enzymes such as PvSAE promises to enable the development of specific inhibitors targeting this class of enzymes. This could be extended to human 9-*O*-SAE, bacterial pathogen 9-*O*-SAEs and viral 9-*O*-SAEs, such as those encoded by specific betacoronaviruses or influenza C.

## Supplementary Material

PDB reference: *Phocaeicola vulgatus* sialic acid acetylesterase, 7pgh

PDB reference: 7pzg

Supplementary Table and Figures. DOI: 10.1107/S2059798322003357/ud5031sup1.pdf

## Figures and Tables

**Table 1:** Macromolecule-production information

Source organism	*Phocaeicola vulgatus*
DNA source	Synthetic
Cloning vector	pET-28 with NcoI and BamHI restriction sites
Expression vector	pET-28
Expression host	*E. coli* BL21(DE3)
Complete amino-acid sequence of the construct produced	MGSSHHHHHHGTAENLYFQGERKYSTFYEQRATLFEELPVTSKDIIFLGNSITNGCEWAELFQNKNVKNRGISGDICMGVYDRLDPIVKGKPAKIFLLIGINDVSRGTSADKIISEISMIVRKIKQESPKTKLYLQSVLPVNDCYGMFNGHTSRWQVVKQINDLLEPLAVKEGVAYIDLYSHFVEKETGKMNPVYTNDGLHLLGKGYLLWRDIVKPYVDQK
Molecular mass (Da)	25191.9
Calculated pI	8.30
Calculated extinction coefficient (*M* ^−1^ cm^−1^)	32890

**Table 2:** Crystallization

	Apo I	Apo II
Method	Sitting drop	Sitting drop
Plate type	48-well MRC Maxi	48-well MRC Maxi
Temperature (K)	293	293
Protein concentration (mg ml^−1^)	30	30
Buffer composition of protein solution	20 m*M* HEPES pH 7.5, 200 m*M* NaCl, 1 m*M* DTT adjusted to pH 7.5 with NaOH	20 m*M* HEPES pH 7.5, 200 m*M* NaCl, 1 m*M* DTT adjusted to pH 7.5 with NaOH
Composition of reservoir solution	18%(*w*/*v*) PEG 3350, 200 m*M* sodium malonate/malonic acid pH 4.5	12.5%(*w*/*v*) PEG 1000, 12.5%(*w*/*v*) PEG 3350, 12.5%(*v*/*v*) MPD, 0.03 *M* sodium nitrate, 0.03 *M* sodium phosphate, 0.03 *M* ammonium sulfate, 0.1 *M* MOPS/HEPES–Na pH 7.5
Volume and ratio of drop	0.8 µl (1:1)	1 µl (1:1)
Volume of reservoir (µl)	100	100

**Table 3:** Data collection and processing Values in parentheses are for the outer shell. For Apo I severe deviation from the Wilson plot was observed, with 10.9% of bins deviating, indicating possible ice rings. For Apo II some deviation from the Wilson plot was observed, with 1.7% of bins deviating. Data resolution cutoff criteria were 〈*I*/σ(*I*)〉 > 1.5 and CC_1/2_ > 0.5.

	Apo I (PDB entry 7pzg)	Apo II (PDB entry 7pzh)
Diffraction source	Beamline I03, DLS	Beamline I03, DLS
Wavelength (Å)	0.97625	0.97624
Temperature (K)	100	100
Detector	EIGER2 XE 16M	EIGER2 XE 16M
Crystal-to-detector distance (mm)	175	175
Rotation range per image (°)	0.1	0.1
Total rotation range (°)	220	220
Exposure time per image (s)	0.01	0.01
Space group	*P*12_1_1	*P*6_1_
*a*, *b*, *c* (Å)	71.3, 90.2, 76.6	93.2, 93.2, 353.6
α, β, γ (°)	90.0, 99.3, 90.0	90.0, 90.0, 120.0
Resolution range (Å)	70.33–1.44 (1.46–1.44)	80.74–2.06 (2.10–2.06)
Total No. of reflections	1144420 (47744)	2186743 (108269)
No. of unique reflections	168248 (7900)	106903 (5267)
Completeness (%)	97.7 (92.8)	100 (100.0)
Multiplicity	6.8 (6.0)	20.5 (20.6)
CC_1/2_	1.0 (0.71)	0.99 (0.54)
〈*I*/σ(*I*)〉	18.3 (1.6)	11.2 (1.8)
*R*_r.i.m._	0.018 (0.416)	0.039 (0.372)
Overall *B* factor from Wilson plot (Å^2^)	17.91	31.11
*L*-test	0.49	0.42

**Table 4:** Structure solution and refinement Values in parentheses are for the outer shell.

	Apo I (PDB entry 7pzg)	Apo II (PDB entry 7pzh)
Resolution range (Å)	70.43–1.44 (1.46–1.44)	80.74–2.06 (2.10–2.06)
Completeness (%)	97.7 (92.8)	100 (100)
No. of reflections, working set	159915 (11313)	101414 (7559)
No. of reflections, test set	8262 (568)	5355 (369)
Final *R*_cryst_	0.18 (0.27)	0.19 (0.26)
Final *R*_free_	0.20 (0.27)	0.23 (0.30)
No. of non-H atoms		
Protein	6390	12810
Ion	4	8
Ligand	44	0
Water	698	260
Total	7136	13078
R.m.s. deviations		
Bond lengths (Å)	0.010	0.009
Angles (°)	1.8	1.6
Average *B* factors (Å^2^)		
Protein	23	41
Ion	32	39
Ligand	37	—
Water	33	35
Ramachandran plot		
Most favoured (%)	97.5	95.8
Allowed (%)	2.3	3.9
Outliers (%)	0	0.1

**Table 5:** Kinetic parameters for the hydrolysis of pNP-Ac catalysed by PvSAE at pH 7.0

Parameter	Value
*K*_m_ (µ*M*)	80 ± 6
*k*_cat_ (s^−1^)	75 ± 1
*k*_cat_/*K*_m_ (m*M* ^−1^ s^−1^)	930 ± 70
