# Supplementary material for: The structure of Phocaeicola vulgatus sialic acid acetylesterase
Source: Acta Crystallogr D Struct Biol. 2022 Apr 26;78(Pt 5):647–57. doi: 10.1107/S2059798322003357 (PMC9063846; doi:10.1107/S2059798322003357)
Supplement: Supplementary file 1 [file d-78-00647-sup1.pdf]

STRUCTURAL BIOLOGY

Volume 78 (2022)

Supporting information for article:

The structure of *Phocaeicola vulgatus* sialic acid acetylesterase

Hannah Scott, Gideon J. Davies and Zachary Armstrong

Table S1 Structurally homologous enzymes to the PvSAE Apo I structure, identified using the DALI server (Holm, 2020).

Description	Source	PBD	Z score	RMSD / Å	L-align	Identity / %
Putative platelet activating factor	*Streptococcus pneumonia*	2hsj	27.4	1.7	194	28
GDSL-like lipase	*Parabacteroides distasonis*	3p94	24.7	2.0	183	25
Putative acylhydrolase	*Bacteroides fragilis*	4ppy	24.2	2.4	190	26
GDSL-like protein	*Bacteroides uniformis*	4iyj	24.2	2.4	190	26
Brain platelet-activating factor acetylhydrolase	*Bos taurus*	3dt9	22.3	2.1	185	20

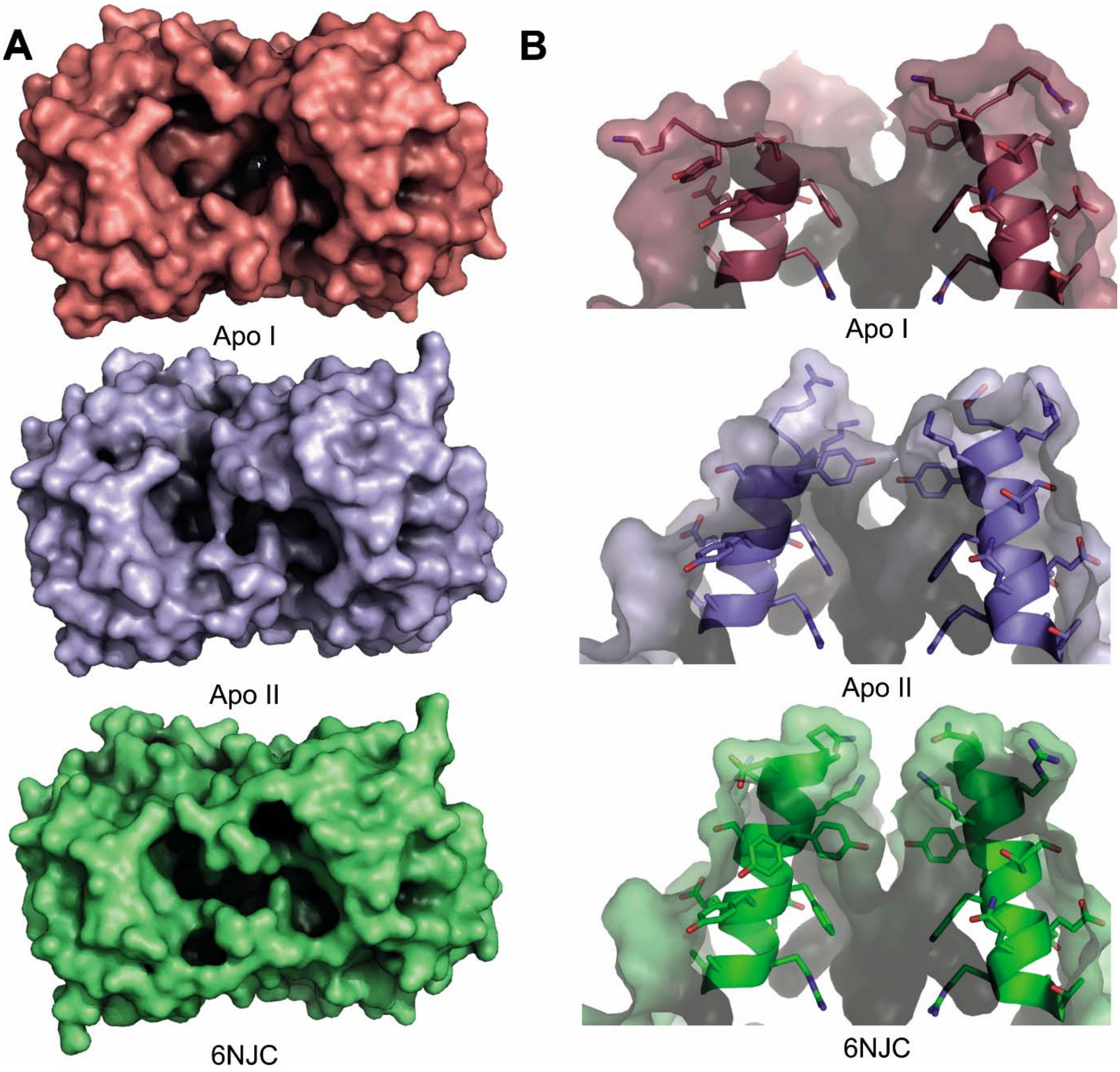

Figure S1 Conformational changes of the N-terminal α-helix. **A**) Surface representation of the Apo I, Apo II and 6njc structures as viewed from above the active site. **B**) The side view of the N-terminal helix from the same structures with residues present in the helix shown in stick representation.

```
PvSAE        1 ------------------------------------------------------------
BfEstA       1 ------------------------------------------------------------
pAF-AH       1 ------------------------------------------------------------
EcNanS       1 MGSSHHHHHHGSNAIISPDYYYVLTVAGQSNAMAYGEGLPLPDREDAPHPRIKQLARFAH

                                                                  Block I
                                                                     *
PvSAE        1 ----------------------ERKYSTFYEQRATLFEELPVTSKDIIFLGNSITNGCE-
BfEstA       1 --MKKIFFLVVILTLSLLCRAQERKYSTFYYQRATLFEELPVTSSDIIFLGNSITNGAE-
pAF-AH       1 --MSGDENPASKPTPVQDVQGDGRWMSLHHRFVADSKDKEP----EVVFIGDSLVQLMHQ
EcNanS      61 THPGGPSCHFNDIIPLTHCPHDVQDMQSYHHPLATNHQTQYGTVGQALHIARKLLPFIPD

                           Block II                             Block III
PvSAE       38 ---WAELFQNKNVKNRGISGDICMGVYDRLD--PIVKGKPAKIFLLIGINDVSRGTSADK
BfEstA      58 ---WAELFKNKHVKNRGISGDICMGVYDRLD--AILKGKPAKIFLLIGINDVSRGTPADT
pAF-AH      55 SEIWRELFSPLHALNFGIGGDSTQHVLWRLENGELEHIRPKIVVVWVGTNNH--GHTAEQ
EcNanS     121 --------------NAGILIVPCCRGGSAFT----AGSEGTYSERHGASHDACRWGTDTP

PvSAE       93 IISEISMIVRKIKQESPKTKLYLQSVLPVNDCYGMFNGHTSRWQVVKQINDLL-EPLAV-
BfEstA     113 IVSRIEMIVRKIKADSPKTKLYLQSVLPVTDHYNMFKGHTSHWQVIPEINKGL-VGLAE-
pAF-AH     113 VTGGIKAIVQLVNERQPQARVVVLGLLPRGQH------PNPLREKNRRVNELVRAALAG-
EcNanS     163 LYQDLVSRTRAALVKNPQNK-FLGVCWMQGEFDLMTSDYASHPQHFNHMVEAFRRDLKQY

                                         Block V
                                         *  *
PvSAE      151 KEGVAYIDLYSHFVEKETGKMNPVYTNDGLHLLGKGYLLWRDIVKPYVDQK---------
BfEstA     171 KEGATYIDLYSHFIDKQTGKMNTTYTNDGLHLLGKGYLKWVEIVKPYIGKK---------
pAF-AH     166 HPRAHFLDADPGFVHSD-GTISHHDMYDYLHLSRLGYTPVCRALHSLLLRLLTQDQGQGG
EcNanS     222 HSQLNNITDAPWFC----GDTTWYWKENFPHAYEAIYGNYQNNILANIIFVDFQQQGARG

PvSAE          ------------------------------------------------------------
BfEstA         ------------------------------------------------------------
pAF-AH     225 APLPEPSP----------------------------------------------------
EcNanS     278 LTNAPDEDPDDLSTGYYGSAYRSPENWTTALRSSHFSSAARRGIISDRFVEAILQFWRER

*Residues in catalytic triad
```

Figure S2 Amino acid sequence alignments for a range of 9-O-SAEs. Alignments include *Phocaeicola vulgatus* PvSAE (ABR41743.1), *Bacteroides fragilis* EstA (WP_100721746.11), *Bos taurus* pAF-AH (PDBID: 3DT6) and *E. coli* NanS (PDBID: 3PT5). Residues forming the catalytic Ser-His-Asp triad are indicated with a star above the amino acid.

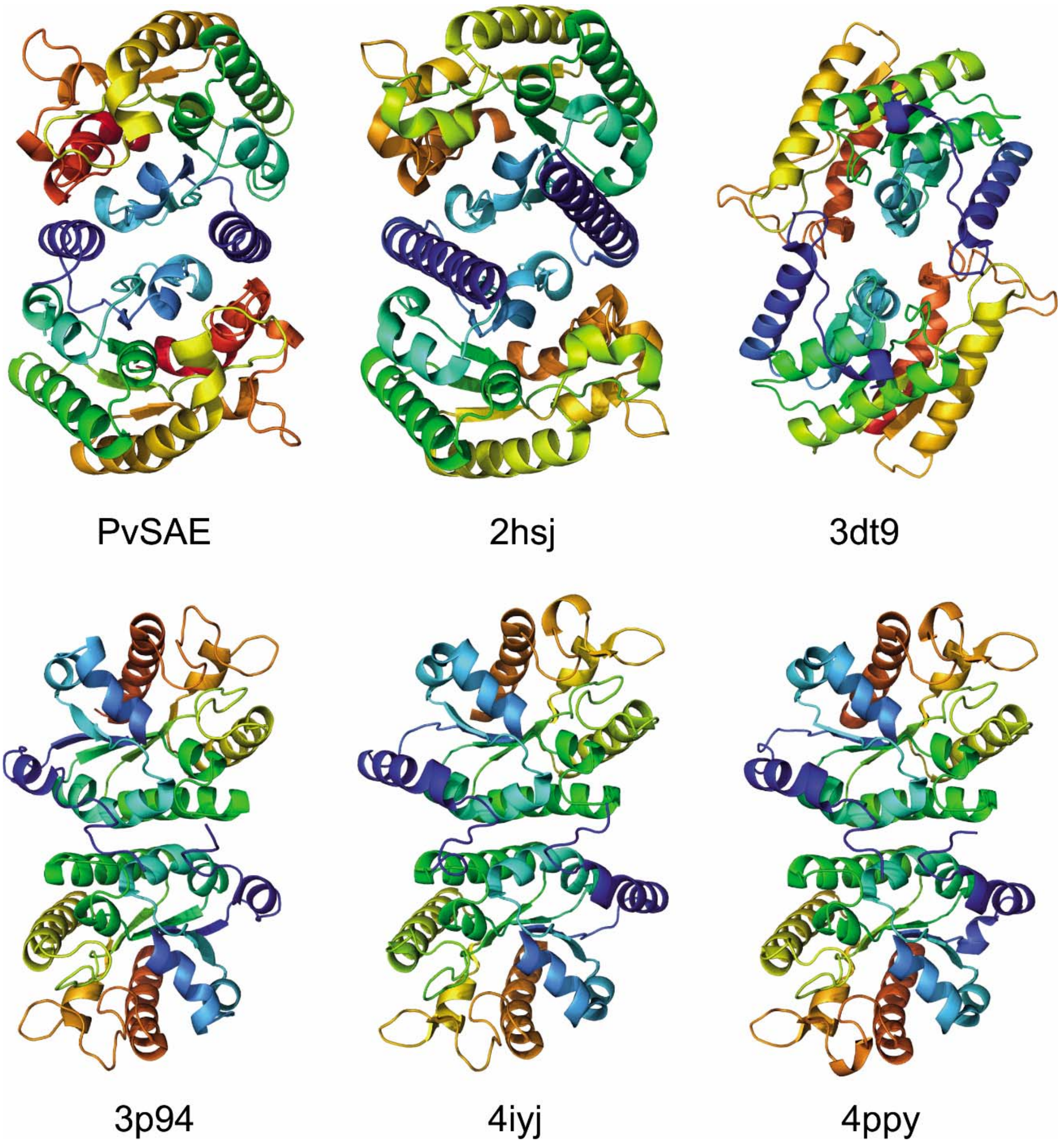

Figure S3 Dimer interfaces of PvSAE and homologs. Dimers are shown in cartoon representation and colored from the N-terminus (purple) to the C-terminus (red).

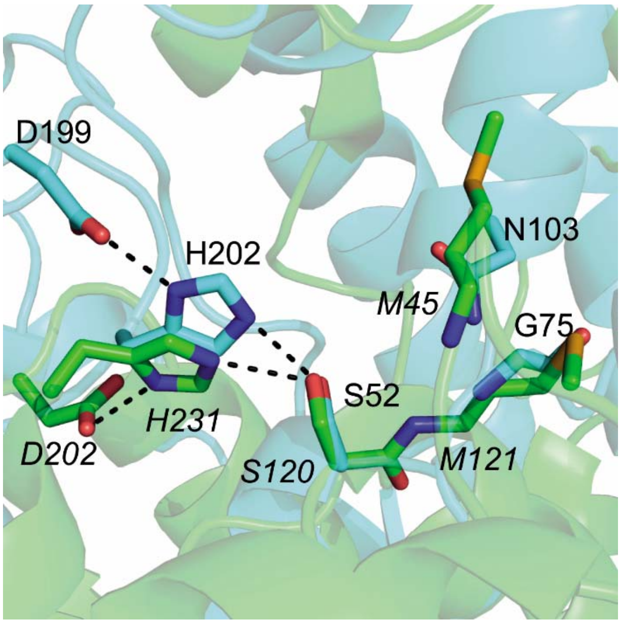

Figure S4 Active site overlay of PvSAE and *S. pneumonia* EstA (PDB: 2uz0)(Kim *et al.*, 2008) with active site residues shown as sticks. *S. pneumonia* EstA residues are labelled in italics and carbons are colored in green, while PvSAE carbons are cyan. Hydrogen bonds between the catalytic triad are shown as black dashed lines.

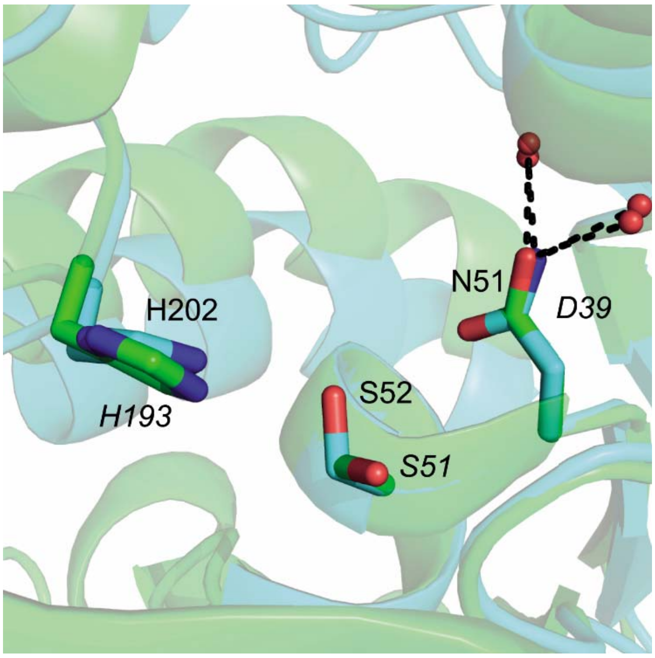

Figure S5 Active site overlay of PvSAE and *S. pneumonia* putative platelet activating factor (PDB-ID: 2hsj) with active site residues shown as sticks. *S. pneumonia* putative platelet activating factor residues are labelled in italics and carbons are colored in green, while PvSAE carbons are cyan. Hydrogen bonds between the catalytic triad are shown as black dashed lines.

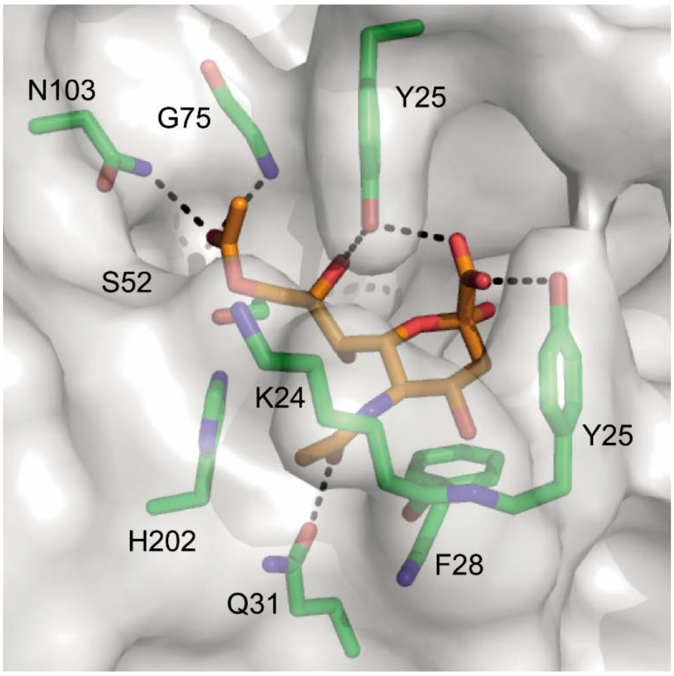

Figure S6 Docking of 9-O-acetylsialic acid into the active site of the Apo II structure. Polar interactions within 3.2 Å are shown as black dashed lines.
